# Computer-Based *De Novo* Designs of Tripeptides as Novel Neuraminidase Inhibitors

**DOI:** 10.3390/ijms11124932

**Published:** 2010-12-01

**Authors:** Zhiwei Yang, Gang Yang, Yuangang Zu, Yujie Fu, Lijun Zhou

**Affiliations:** 1 Key Laboratory of Forest Plant Ecology, Ministry of Education, Northeast Forestry University, Harbin 150040, China; E-Mails: yzws-123@163.com (Z.Y.); fuyujie1967@yahoo.com.cn (Y.F.); zlj_1008@yahoo.com.cn (L.Z.); 2 Dalian Institute of Chemical Physics, Chinese Academy of Sciences, Dalian 116023, China

**Keywords:** docking, influenza, neuraminidase inhibitors, tripeptides, H-bonds, *de novo* drug designs

## Abstract

The latest influenza A (H1N1) pandemic attracted worldwide attention and called for the urgent development of novel antiviral drugs. Here, seven tripeptides are designed and explored as neuraminidase (NA) inhibitors on the structural basis of known inhibitors. Their interactions with NA are studied and compared with each other, using flexible docking and molecular dynamics simulations. The various composed tripeptides have respective binding specificities and their interaction energies with NA decrease in the order of FRI > FRV > FRT > FHV > FRS > FRG > YRV (letters corresponding to amino acid code). The Arg and Phe portions of the tripeptides play important roles during the binding process: Arg has strong electrostatic interactions with the key residues Asp151, Glu119, Glu227 and Glu277, whereas Phe fits well in the hydrophobic cave within the NA active site. Owing to the introduction of hydrophobic property, the interaction energies of FRV and FRI are larger; in particular, FRI demonstrates the best binding quality and shows potential as a lead compound. In addition, the influence of the chemical states of the terminal amino acids are clarified: it is revealed that the charged states of the *N*-terminus (NH_3_^+^) and *C*-terminus (COO^−^) are crucial for the tripeptide inhibitory activities and longer peptides may not be appropriate. In addition, the medium inhibiting activity by acetylation of the *N*-terminus indicates the possible chemical modifications of FRI. Experimental efforts are expected in order to actualize the tripeptides as potent NA inhibitors in the near future.

## 1. Introduction

At present, influenza is probably the most serious pandemic threat to human health [1–3]. The influenza virus also causes severe morbidity and mortality in poultry as a result of co-infection with other pathogens [4]. Zanamivir and oseltamivir (known as Tamiflu) are two known anti-influenza drugs that have been widely used in the chemoprophylaxis and treatment of influenza and stockpiled in preparation for pandemic outbreak [4–7]. However, cases of zanamivir or/and oseltamivir resistant strains have been reported [8–10]. It thus becomes very urgent to develop novel and efficient anti-influenza drugs in order to prevent and treat influenza infections [2].

Neuraminidase (NA) is a major surface glycoprotein of influenza virus that plays a crucial role in the release of new viral particles [11]. The inhibition of NA will delay the release of progeny virions from infected host cell and thus allow the host immune systems sufficient time to clear them [12]. The active sites of NAs are highly conserved across different sub-types of influenza viruses, especially for N2 and N9 sub-types (nearly identical) [2,13,14]. Accordingly, NA is an ideal target for the rational designs of next-generation anti-influenza drugs [15]. Consistent efforts have been devoted to the development of NA inhibitors (NAIs), using the crystal structures of N9 sub-type NA proteins [2,16–19]. Zanamivir and oseltamivir are two representative NAIs that have proven to be successful and have been commercialized for human use [5,20,21].

Recently, peptides against influenza viruses have shown potential as therapeutic agents [22–25]. It was found that the peptides RRKKAAVALLPAVLLALLAP, CNDFRSKTC and NDFRSKT exhibit antiviral properties and inhibit viruses’ attachments to cellular receptors [22,25]. In addition, the 12-mer peptides (54-N1 and 69-N2) display broad-spectrum inhibitory activities against influenza virus through interactions with the NA proteins [23]. However, these peptides are only partially docked into the NA active sites and will not form compact binding complexes [23]; moreover, they are not facile to synthesize and commercialize, owing to their relatively large molecular sizes. *In vitro* experiments revealed that the inhibiting activity of peptide NDFRSKT is clearly higher than that of peptide CNDFRSKTC [25], where the contained tripeptide FRS may act as the active center. Accordingly, it is of high urgency to discover novel, shorter peptides as lead compounds of the next generation anti-influenza agents.

Although peptides have limited *in vivo* bioavailability, this does not hamper the extensive exploitation of peptide-based drugs [26–30]. Some of the top 100 best-selling drugs approved by the FDA are peptides [29]. Especially, tripeptides have played an important role in biological processes and drug designs [31], of which glutathione (GSH) is probably the most familiar to us [32]. There are reports that tripeptides contribute a lot to clinical research, such as thrombin [33], HIV protease [34], HCV protease [35] and immune systems [36]. On the basis of the evaluations of oseltamivir carboxylate (the active form of oseltamivir) and 4-(*N*-acetylamino)-5-guanidino-3-(3-pentyloxy) benzoic acid (BA) and three-dimensional information about the NA active site [2,14,37,38], tripeptides FRG, FRV, FHV, YRV, FRT, FRS and FRI were designed as NA inhibitors (Figure S1). The seven tripeptides, optimized with density functional methods, were docked into the NA active site, respectively, and their interaction mechanisms were then studied by explicitly solvated molecular dynamics (MD) simulations. It was found that FRI has the largest interaction energy and matches satisfactorily in the NA active site, which throws new light on the *de novo* designs of NA inhibitors. Around physiological pH values, the *N*-terminus and *C*-terminus of FRI are charged, in the forms of NH_3_^+^ and COO^−^, respectively. How the chemical states of the termini influence the inhibitor binding at the NA active site is also an interesting topic that requires attention. In addition, the deprotonation and acetylation at the *N*-terminus (-NH_2_ and -NHCOCH_3_), as well as the amidation at the *C*-terminus (-CON(CH_3_)_2_) were considered, with their structures optimized at the same level of theory. The interaction mechanisms of the three structures with NA were also studied by explicitly solvated flexible docking and MD simulations. The present results can guide synthetic and medicinal chemists to discover potent peptide-based antiviral drugs.

## 2. Results and Discussion

**Figure 1.**
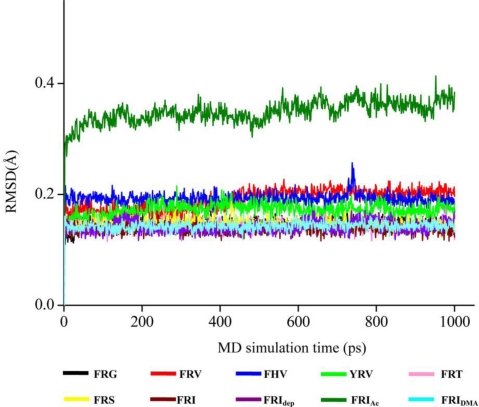
The time-evolution backbone-atom root mean square deviations (RMSD) of protein structures in the tripeptide-NA complexes.

**Figure 2.**
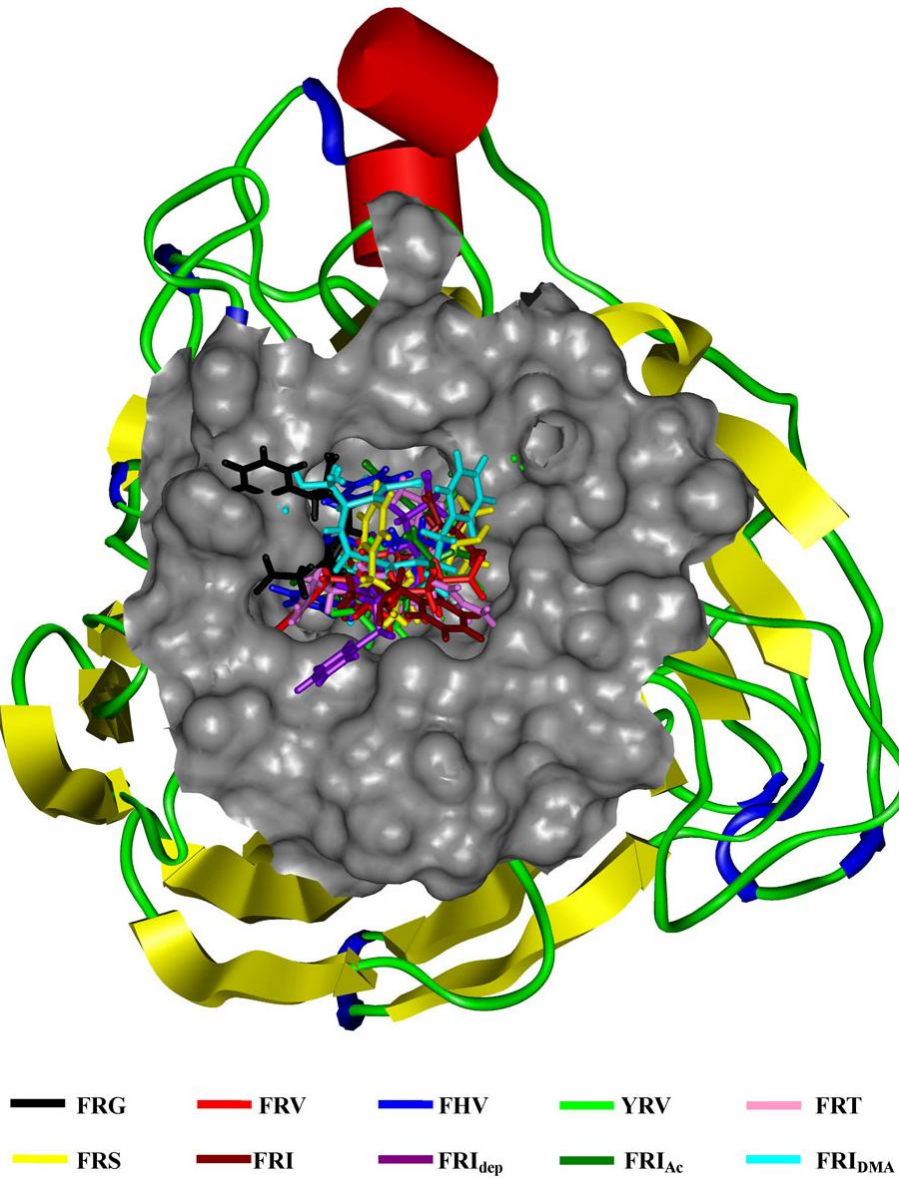
The tripeptides superposed at the NA active site. The Connolly surfaces of the NA active-site (in grey) are created using the InsightII 2005 scripts. The tripeptides are represented by stick models. Ribbon colors: Helices (including α-, 3_10_- and π-helix), hydrogen-bonded turns, extended strands and random coils are in red, blue, yellow and green, respectively.

As the backbone-atom root-mean-square deviations (RMSD) in Figure 1 indicate, all the tripeptide-NA complexes that have been energy-minimized remain stable throughout the 1.0 ns MD simulations, consistent with the previous MD results of other NA inhibitors [37–41]. Accordingly, the geometric and energetic analyses are made on the average structures of 500∼1000 ps MD trajectories, where the docked complexes are already at equilibrium. The superposed structures in Figure 2 show that the seven tripeptides are in close space at the NA active site, in terms of favorable interaction energies and geometrical matching qualities. It means that these tripeptides occupy the identical binding pocket of the NA protein. However, their binding poses differ somewhat from each other, which will be discussed in the following sections.

### 2.1. The Tripeptides FRG and FRV as the NA Inhibitors

On basis of the structures of known anti-influenza virus drugs such as oseltamivir carboxylate and BA [2,14,37,38] and the electrostatic, steric and lipophilic characteristics of NA active sites [1,2,13,23,37,38,42,43], tripeptide FRG was first designed as NA inhibitor. That is, the simplest amino acid Gly is at its *C*-terminus, which is expected to orient towards the Arg triad (Arg118, Arg292 and Arg371). The guanidino group of FRG is assumed to direct to the acidic sub-site consisting of residues Glu119, Asp151 and Glu227. In order to fit the hydrophobic cave of the NA active site, the hydrophobic interactions are introduced at the *N*-terminus using Phe.

**Figure 3.**
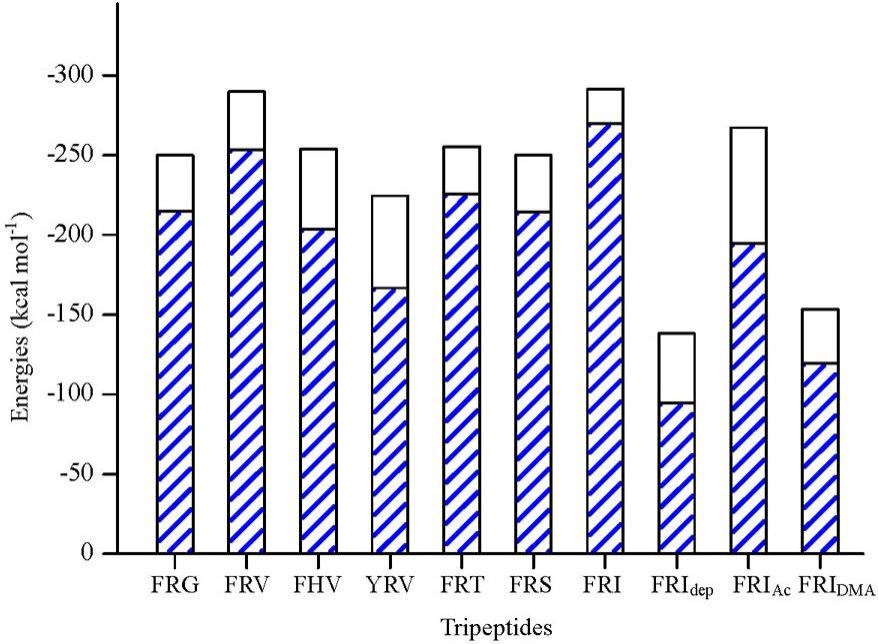
The electrostatic (*E*_ele_, blue sparse area) and total interaction energies (*E*_inter_) between NA protein and various tripeptides.

**Figure 4.**
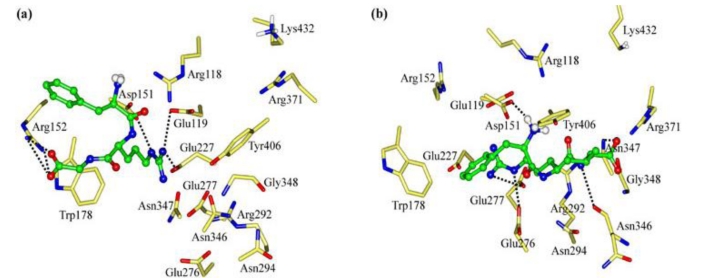
Views of the binding modes of the NA active-site residues with (**a**) FRG and (**b**) FRV. Key residues are represented by stick models. Tripeptides are represented by ball and stick models. The important H-bonds are labeled as dashed black lines.

The interaction energy (*E*_inter_) of FRG with NA is calculated at −249.83 kcal mol^−1^, where the electrostatic rather than vdW interactions are found to play a dominant role (Figure 3). As Figure 4a shows, the carboxyl group of FRG has three H-bonds with the positively charged guanidino group of residue Arg152. The guanidino group of FRG forms ionic interactions with the negatively charged carboxyl groups of residues Glu119, Asp151 and Glu227, with one H-bond formed with each residue. The electrostatic contributions (*E*_ele_) of residues Glu119, Asp151, Arg152 and Glu227 amount to −92.93, −132.39, −37.21 and −63.39 kcal mol^−1^, respectively (Table S1). Nonetheless, the benzene group of FRG somewhat flips out from the NA active site. The lack of sidechains in Gly (H atoms) does not match with the hydrophobic portion of the NA active site. Accordingly, the *C*-terminus of FRG (Gly) is improved by the G→V mutation.

Compared with Gly, the sidechain of Val is more suited to the NA active site (Figure 4). The interaction energy (*E*_inter_) between FRV and NA is equal to −289.88 kcal mol^−1^. The FRV has a value 40 kcal mol^−1^ larger than FRG (Figure 3). Similar to FRG, the electrostatic rather than vdW interactions dominate during the binding process. The FRV carboxyl group orients towards the Arg triad (Arg118, Arg292 and Arg371) of the NA active site (Figure 4b). The guanidino group of FRV shows strong electrostatic effects with residues Glu119, Asp151, Glu227, Glu276 and Glu277, with the intergrowth of one H-bond to each of residues Glu276 and Glu277. The corresponding electrostatic contributions (*E*_ele_) are calculated at −73.28, −125.36, −68.08, −103.29 and −101.73 kcal mol^−1^, respectively (Table S1). In addition, the amino group at the *N*-terminus of FRV forms one H-bond with residue Asp151. The benzene group of FRV conduces to the hydrophobic contacts with the NA active site. Accordingly, the FRV poses in the NA active site, with a similar manner as the cases of current NA drugs (oseltamivir, zanamivir and peramivir) [2,5,14,38,42,44]. Compared with FRG, more preference of FRV has been observed. The Arg and/or Phe portions of the tripeptides play crucial roles during the binding process, which will be clarified in the following discussions.

### 2.2. The Roles of the Arg and Phe Portions in the Tripeptides

**Figure 5.**
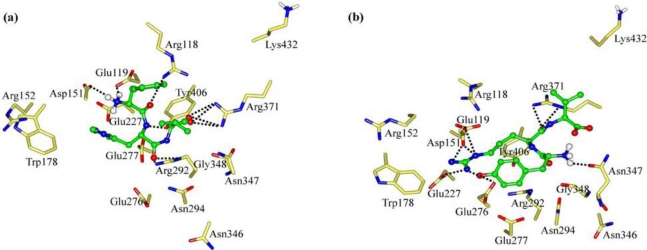
Views of the binding modes of the NA active-site residues with (**a**) FHV and (**b**) YRV. Key residues are represented by stick models. Tripeptides are represented by ball and stick models. The important H-bonds are labeled as dashed black lines.

In order to clarify the roles of the Arg and Phe portions in the tripeptide FRV, another two tripeptides FHV and YRV were designed as NA inhibitors. The interaction energy (*E*_inter_) of FHV with NA is calculated to be −254.00 kcal mol^−1^ (Figure 3). As a result of the disappearance of the Arg portion (R→H mutation), the interaction energy (*E*_inter_) of FHV is about 36 kcal mol^−1^ lower than that of FRV. The His portion of FHV does not form any H-bond with the active-site residues and rather moves out of the active-site pocket (Figure 5a). In the meantime, the charge transfers of FHV with residues Glu119, Asp151, Glu227, Glu276 and Glu277 decrease, in contrast to the case of FRV, especially residues Glu276 and Glu277 (Tables S1 and S2). Accordingly, the Arg portion is crucial to FRV and responsible for the lower interaction energy of FHV.

For YRV, the interaction energy (*E*_inter_) with NA equals −224.51 kcal mol^−1^ and is less than either of the tripeptides FRV and FHV (Figure 3). As shown in Figure 5b, the carboxyl group of YRV deviates from the Arg triad (Arg118, Arg292 and Arg371), which is quite different from the situations of FRV and FHV. Compared with FRV, the electrostatic interactions (*E*_ele_) of the YRV guanidino group with residues Glu276 and Glu277 remarkably reduce to −52.17 and −69.65 kcal mol^−1^, respectively (Table S2). Accordingly, the increase of polarity due to the F→Y mutation (FRV to YRV) is unfavorable for the binding process. To summarize, the Arg and Phe portions of the tripeptides, especially the latter, are crucial to the binding process and should be reserved in the design of tripeptide inhibitors.

### 2.3. The Improvement of FRV-Based NA Inhibitors

**Figure 6.**
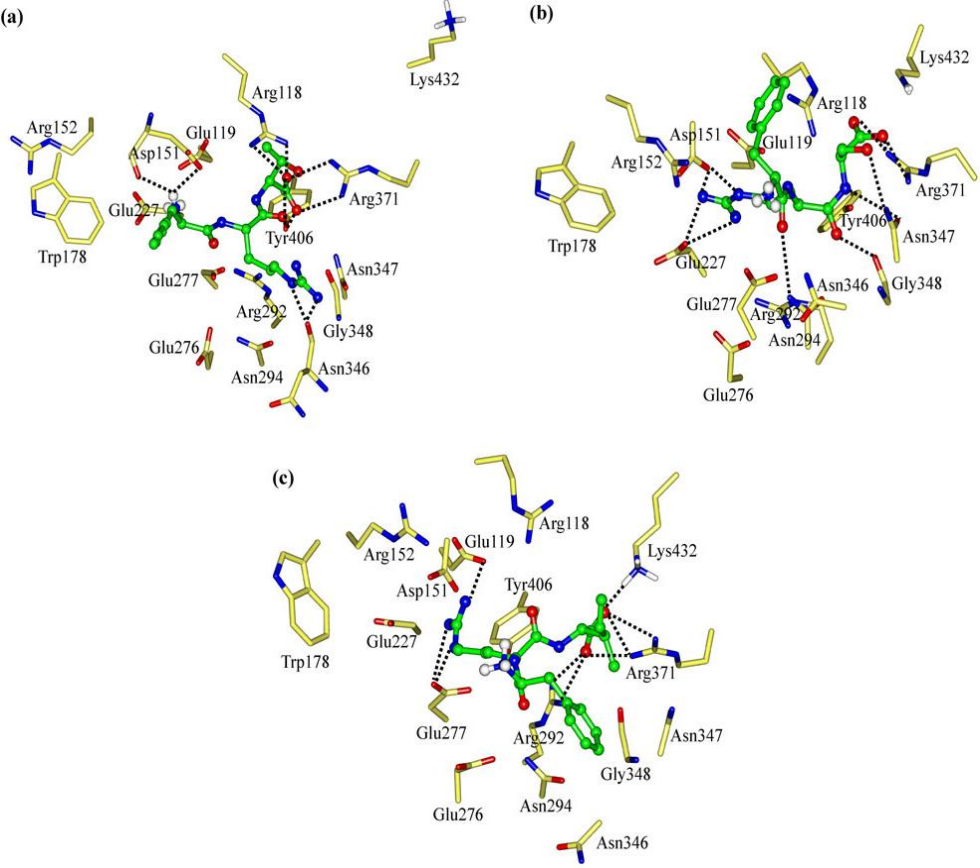
Views of the binding modes of the NA active-site residues with (**a**) FRT, (**b**) FRS and (**c**) FRI. Key residues are represented by stick models. Tripeptides are represented by ball and stick models. The important H-bonds are labeled as dashed black lines.

Among the four tripeptides discussed in Sections 2.1 and 2.2, FRV has the largest interaction energy with the NA protein and its binding pose is similar to those of current NA drugs [2,5,14,38,42,44]. In addition, the Arg and Phe portions of FRV play important roles during the binding process. Accordingly, the improvement of tripeptide-based NA inhibitors was next centered on the mutations of the *C*-terminus amino acid Val. In this way, tripeptides FRT, FRS and FRI were designed as NA inhibitors. Their binding at the NA active site is shown in Figure 6.

Owing to the addition of hydrophilic property by the V→T mutation, the interaction energy (*E*_inter_) of FRT with NA drops to −255.45 kcal mol^−1^ (Figure 3). As Figures 4b and 6a show, the maximal binding differences between FRT-NA and FRV-NA are in that the FRT guanidino group orients towards Asn346 with two H-bonds formed. In addition, the FRT benzene group is somewhat out of the NA active site. Similar to FRV, the charge transfer interactions are observed between FRT and residues Glu119, Asp151, Glu227, Glu276 and Glu277 (Table S3). However, their electrostatic contributions (*E*_ele_) decrease remarkably. Accordingly, FRV instead of FRT suits the NA active site better.

The tripeptide FRS is more hydrophilic than FRV (V→S mutation) and meanwhile its spatial size is less than that of FRT. The interaction energy (*E*_inter_) of FRS with NA is calculated at −250.04 kcal mol^−1^, somewhat less than those of FRV and FRT (Figure 3). The binding pose of FRS at the NA active site is shown in Figure 6b. The FRS carboxyl group is stabilized by residue Arg371, with the formation of three H-bonds. The FRS guanidino group forms two H-bonds with each of residues Asp151 and Glu227. Compared with FRV, the tripeptide FRS has less electrostatic interactions with residues Glu119, Asp151, Glu227, Glu276 and Glu277. Especially the electrostatic contribution (*E*_ele_) of residue Glu276 is merely equal to −45.66 kcal mol^−1^ (Table S3). More importantly, the orientation of the FRS benzene group deviates somewhat from the NA active site. It indicates that FRS does not better suit the NA active site than FRV.

Owing to the increase in spatial size and hydrophobic property introduced by the V→I mutation, FRI moves closer to the NA active-site pocket (Figure 6c). The interaction energy (*E*_inter_) of FRI equals −291.56 kcal mol^−1^ and is larger than any of the above six tripeptides (Figure 3). Similarly, the electrostatic rather vdW interactions play a dominant role during the binding process. The FRI carboxyl group forms ionic interactions with residues Arg292, Arg371 and Lys432, with two, three and one vigorous H-bonds, respectively (Figure 6c). The electrostatic energies (*E*_ele_) from residues Arg371 and Lys432 amount to −43.88 and −29.62 kcal mol^−1^, respectively (Table S3), while these two values are very slight in the case of FRV. The FRI guanidino group shows strong interactions with residues Glu119 and Glu277, with one and two H-bonds formed, respectively. The FRI benzene group fits perfectly with the hydrophobic cave of the NA active site. FRI has complementary properties against the geometrical and biophysical environment of the NA active site, which can also be observed in the cases of current potent NA drugs; e.g., oseltamivir, zanamivir and peramivir [2,5,14,38,42,44].

### 2.4. The Chemical States of the Termini in Tripeptide FRI

For all the seven tripeptides, the electrostatic interactions play a dominant role during their binding processes (Figure 3), which is consistent with the current NA drugs [2,5,14,38,42,44]. The carboxyl groups of the seven tripeptides, except for FRG and YRV, have strong electrostatic interactions with the Arg triad (Arg118, Arg292 and Arg371) and should be fully considered in rational drug designs. The residues Asp151, Glu119, Glu227 and Glu277 of the NA protein contribute greatly in all the seven cases, see the data in Tables S1–S4. In fact, these four residues of the NA protein have already received enough attention from rational drug designs [2,14,42]. The catalytic residue Asp151 is crucial to NA function and the three glutamic acid residues (Glu119, Glu227 and Glu277) are important to stabilize the NA active sites [1,45]. With the low toxicity and viral resistance, the tripeptides throw new light on the rational designs of novel anti-influenza drugs [23]. In particular, the interaction energy (*E*_inter_) of FRI with NA is equal to −291.56 kcal mol^−1^ and the largest among the seven tripeptides, which is also much larger than that of 4-(*N*-acetylamino)-5-guanidino-3-(3-pentyloxy) benzoic acid (BA, −160.64 kcal mol^−1^) [37]. Accordingly, the tripeptide FRI shows great potential as an ideal lead compound.

**Figure 7.**
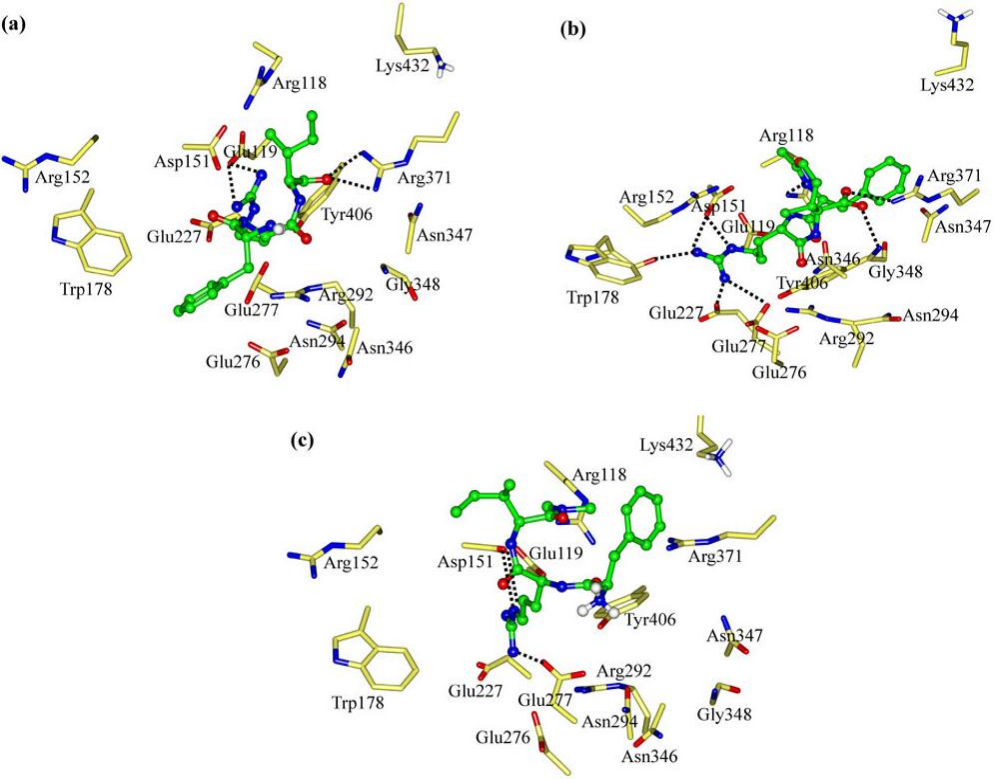
Views of the binding modes of the NA active-site residues with (**a**) FRI_dep_, (**b**) FRI_Ac_ and (**c**) FRI_DMA_. Key residues are represented by stick models and tripeptides are by ball and stick models. The important H-bonds are labeled as dashed black lines.

As mentioned in Section 1, the *N*-terminus of FRI is in the NH_3_^+^ form around physiological pH values (Figure 4a). Besides, the deprotonated (-NH_2_) and acetylated (-NHCOCH_3_) forms are also considered and designated as FRI_dep_ and FRI_Ac_, respectively. The deprotonation causes the FRI_dep_ benzene group to move out of the NA active-site pocket (Figure 7a), with the interaction energy being obviously reduced (Figure 3). The interaction energy (*E*_inter_) between FRI_dep_ and NA is summed to −138.22 kcal mol^−1^; less than half of the normal state (NH_3_^+^). The deprotonation also decreases the electrostatic interactions with the NA active-site residues; e.g., residue Glu277, whose electrostatic contribution (*E*_ele_) drops sharply from −108.43 to −19.89 kcal mol^−1^ (Table S4). In addition, the H-bonds are merely four: two between the FRI_dep_ carboxyl group and residue Arg371 and two between the FRI_dep_ guanidino group and residue Glu119 (Figure 7a). Accordingly, the depronation of the *N*-terminus NH_3_^+^ group is not favored for the inhibiting activities of the tripeptides.

When the *N*-terminus of FRI is protected by acetylization (-NHCOCH_3_), the interaction energy (*E*_inter_) with the NA protein is calculated to be −267.32 kcal mol^−1^. It indicates that the acetylization also decreases the inhibiting activity but more slightly than the deprotonation (Figure 3). Compared with the normal state (NH_3_^+^), the acetylization causes the carboxyl group to move towards residue Arg371, with the simultaneous formation of one strong H-bond (Figure 7b). The electrostatic energy (*E*_ele_) of FRI_Ac_ with residue Arg371 increases to −62.66 kcal mol^−1^ (Table S4). However, the ionic interactions with residue Lys432 do not exist anymore. At the same time, the FRI_Ac_ guanidino group has less polar contacts with residues Glu227 and Glu277, with one H-bond formed with each of them. Accordingly, FRI rather than FRI_Ac_ and FRI_dep_ matches satisfactorily with the NA active site. The decrease of interaction energies by the acetylization is in good agreement with experiment data that long peptide chains may not form compact binding complexes with the NA receptors [22,23,25]. Nonetheless, FRI_Ac_ still displays reasonable space orientation at the NA active site and medium inhibiting activity, probably due to that the lost electrostatic binding has been compensated by the hydrophobic effects between its acetyl-CH_3_ and the small pocket of NA active site. This indicates the possible prospects of chemical modifications.

Similar to the *N*-terminus, the *C*-capped FRI is designed as FRI_DMA_, in order to explore the roles of the *C*-terminus chemical states during the binding processes. As shown in Figure 3, the *C*-terminus capping (-CON(CH_3_)_2_) causes the interaction energy (*E*_inter_) of FRI_DMA_ with NA to reduce to −153.29 kcal mol^−1^, about half of the normal state (COO^−^). Compared with FRI, the Phe and Ile portions of FRI_DMA_ nearly move out of the active-site pocket. Only the guanidino group of FRI_DMA_ forms two H-bonds with Asp151 and one H-bond with Glu277 (Figure 7c). Moreover, the ionic interactions with residues Arg371 and Lys432 are dissolved as well (Table S4). Accordingly, the capping of *C*-terminus (-CON(CH_3_)_2_) disrupts the tripeptide interactions with the NA active sites, in good agreement with previous reports that the charged carboxyl groups (COO^−^) are important for anchoring inhibitors in the NA active sites by strong electrostatic interactions [37,38,43,46]. It further indicates that the long peptides may not be suitable to be designed as NA inhibitors.

## 3. Computational Methods

The docking and molecular dynamics (MD) simulations were performed with the different modules implemented under InsightII 2005 software package [47] on Linux workstations.

### 3.1. System Preparations

The N9 sub-type neuraminidase (NA) crystal structure (PDB code: 1F8B) was recovered from the RCSB Protein Data Bank [18]. For convenience, it is named NA throughout this work. The calcium ion (Ca^2+^) and crystal water molecules near the active site were retained in the protein structure. The hydrogen atoms were then added on basis of the expected charge distribution of amino acids at physiological pH values [37,38,46,47]. The particular protonation states of residues with titratable groups were taken with the aid of the Biopolymer module and manual verification [18,37,38,40,47]. Note that the sidechain of residue Asn294 in NA was rotated so that its Oδ1 and Nδ2 atoms of the amide group form H-bonds with the nearby Ala246 O and Arg292 Nɛ2 atoms, which will improve the agreement with the overall crystal structure [40]. The NA structure was then neutralized with chloride anions [37,38,43,46,48]. The conjugated gradient algorithm was used to optimize the NA structure (Discover 3.0 module), with the consistent-valence force-field (CVFF). The convergence criterion was set to 0.01 kcal mol^−1^ Å^−1^.

All the tripeptides (Figure S1) were optimized with density functional methods [49,50], and the details can be found in Supplementary Material.

### 3.2. Flexible Docking

The docking simulations were performed by the protocol used in our previous works [37,38,43,46]. The Binding-site module was used to identify the NA active site. Then, the advanced docking program Affinity, combining Monte Carlo (MC) and simulated annealing (SA) methods, was used to determine the optimal orientations of the tripeptides at the NA active sites [51]. A feature for the semi-flexible method is that the ligand and the defined active-site residues were allowed to move freely whereas the rest of proteins were held rigid during the docking process. The potential function was assigned using the CVFF force-field and the non-bonded interactions were described by the Cell-Multipole approach. The solvent effects were considered by solvating the complexes in a large sphere of TIP3P water molecules [52] with the radius of 35.0 Å. Chloride anions were added to neutralize the docked systems [37,38,43,46,48]. The docked complexes were selected on basis of interaction energies and geometrical matching qualities. The selected complexes were further energy-minimized using the conjugated gradient method until converged to 0.01 kcal mol^−1^ Å^−1^.

### 3.3. Molecular Dynamics (MD)

The MD simulations were performed on the energy-minimized docked complexes, using the CVFF force-field in Discover 3.0 module. The canonical ensemble (NVT) was employed. The simulation temperature is 300.0 K (normal temperature), which was controlled by the Langevin thermostat [53]. The integration of the classical equations of motion was achieved using the Verlet algorithm. During the MD simulations, the inhibitors plus a surrounding sphere of 10.0 Å were allowed to move freely whereas the rest were held rigid, consistent with previous works [37,38,46]. The MD trajectories were generated using a 1.0-fs time step for a total of 1000 ps, saved at 1.0-ps intervals. The interaction energies of tripeptides with NA and the respective residues at the NA active site were calculated by the Docking module, over the average structures of 500∼1000 ps MD trajectories [51].

## 4. Conclusions

In this work, a series of tripeptides were explored as potential neuraminidase (NA) inhibitors. Their interactions with the NA protein were then studied by flexible docking and molecular dynamics (MD) simulations. In addition, the influences were clarified for the chemical states of terminus amino acids. This study will guide synthetic and medicinal chemists to discover potent tripeptides as novel anti-influenza virus drugs.

Based on the structures of known NA inhibitors and the properties of NA active sites, FRG, FRV, FHV, YRV, FRT, FRS and FRI were successively designed as NA inhibitors. Details of the binding specificity for each of the seven tripeptides at the NA active site are given in the text. The interaction energies decrease in the order of FRI (−291.56 kcal mol^−1^) > FRV (−289.88 kcal mol^−1^) > FRT (−255.45 kcal mol^−1^) > FHV (−254.00 kcal mol^−1^) > FRS (−250.04 kcal mol^−1^) > FRG (−249.83 kcal mol^−1^) > YRV (−224.51 kcal mol^−1^). The Arg and Phe portions of the tripeptide-based NA inhibitors are crucial to the binding process: The former has strong electrostatic interactions with residues Asp151, Glu119, Glu227 and Glu277, in good agreement with the data of commercial NA inhibitors; while the latter perfectly fits the hydrophobic cave of the NA active site. Moreover, the addition of proper hydrophobicity facilitates the interactions. Among the seven tripeptides, FRI best matches the NA active site and has the largest interaction energy, obviously superior to the potential drug 4-(*N*-acetylamino)-5-guanidino-3-(3-pentyloxy)benzoic acid (−160.64 kcal mol^−1^). Accordingly, it is an ideal lead compound for the designs of tripeptide-based NA inhibitors.

The deprotonation or acetylization of the *N*-terminus NH_3_^+^ group, as well as the amidation of the *C*-terminus COO^−^ group causes reduction of the binding qualities. Accordingly, the charged forms of the *N*- and *C*-termini (*i.e.*, NH_3_^+^ and COO^−^) are crucial for the tripeptide inhibitory activities. The longer peptide chains may not form compact binding complexes with the NA protein. Nonetheless, FRI_Ac_ still shows reasonable spatial orientation at the NA active site and medium inhibiting activity, indicating the possible chemical modifications. We believe that this work will arouse the interest of experimental aspects and result in potent tripeptide-based NA inhibitors in the near future.

## Supplemental Material

The details of density functional methods and interaction energies of tripeptides with the NA active-site residues can be found as supplemental material.

## Supplementary Materials

### 1. Density Functional Calculations

As shown in Figure S1, seven tripeptides FRG, FRV, FHV, YRV, FRT, FRS and FRI were designed, which were then optimized with B3LYP density functional methods within Gaussian03 software [49,54–59]. The standard 6-31G(d,p) basis set was used [50,60]. The *N*- and *C*-termini of these tripeptides are protonated and depronated around the physiological pH values; *i.e*., in the NH_3_^+^ and COO^−^ forms, respectively. In addition, three other states were considered for the tripeptide FRI: the *N*-terminus is neutral (-NH_2_, see FRI_dep_ in Figure S1h), the *N*-terminus is acetylated (-NHCOCH_3_, see FRI_Ac_ in Figure S1i) and the *C*-terminus is amidated (-CON(CH_3_)_2_, see FRI_DMA_ in Figure S1j), respectively. The three structures were also optimized with B3LYP/6-31G (d,p) methods. Frequency calculations at the same level of theory were performed for all the above structures, confirming that they are stable minima on their respective potential energy surfaces (PES) [50].

Figure S1. Optimized tripeptide structures at B3LYP/6-31G(d,p) level of theory: (**a**) FRG; (**b**) FRV; (**c**) FHV; (**d**) YRV; (**e**) FRT; (**f**) FRS; (**g**) FRI; (**h**) FRI_dep_; (**i**) FRI_Ac_ and (**j**) FRI_DMA_.

**Table S1.** The vdW, electrostatic and total interaction energies (*E*_vdW_, *E*_ele_ and *E*_inter_) between FRG, FRV and NA active site residues*ab*.

	**FRG-NA**			**FRV-NA**		

Residue	*E* _vdW_	*E* _ele_	*E* _inter_	*E* _vdW_	*E* _ele_	*E* _inter_
Glu119	−1.23	−93.23	−94.46	−1.39	−73.28	−74.67
Ile149	−1.18	−9.19	−10.37	−	−	−
His150	−8.32	−6.59	−14.91	−	−	−
Asp151	−2.86	−132.39	−135.25	−5.32	−125.36	−130.68
Arg152	−2.55	−37.21	−39.76	−	−	−
Trp178	−	−	−	−3.11	−1.33	−4.44
Glu227	−1.02	−63.39	−66.41	−0.97	−68.08	−69.05
Ala246	−	−	−	−5.62	−0.30	−5.92
Thr247	−	−	−	−3.07	−2.93	−6.00
Glu276	−0.18	−37.08	−37.26	−0.24	−103.29	−103.53
Glu277	−1.32	−66.47	−67.79	−0.02	−101.73	−101.75
Asn347	−2.34	−6.17	−8.51	−4.11	−20.27	−24.38

a Energy units in kcal mol^−1^;

b *E*_inter_ < −4.00 kcal mol^−1^ are given.

**Table S2.** The vdW, electrostatic and total interaction energies (*E*_vdW_, *E*_ele_ and *E*_inter_) between FHV, YRV and NA active-site residues*a, b*.

	**FHV-NA**			**YRV-NA**		

Residue	E *_vdW_*	E *_ele_*	E *_inter_*	E *_vdW_*	E *_ele_*	E *_inter_*
Arg118	−2.58	−13.71	−16.29	−	−	−
Glu119	−0.07	−57.24	−57.31	−	−	−
Ile149	−4.16	−1.51	−5.67	−	−	−
Asp151	−5.74	−57.83	−63.57	−0.48	−126.92	−127.40
Glu227	−0.36	−14.15	−14.51	−0.88	−63.89	−64.77
Glu276	−	−	−	−2.33	−52.17	−54.50
Glu277	−2.80	−5.07	−7.87	−1.12	−69.65	−70.77
Arg292	−3.49	−28.50	−31.99	−	−	−
Asn347	−	−	−	−2.70	−6.33	−9.03
Arg371	1.72	−84.84	−83.12	−	−	−
Lys432	−0.09	−4.02	−4.11	−	−	−

a Energy units in kcal mol^−1^;

b *E*_inter_ < −4.00 kcal mol^−1^ are given.

**Table S3.** The vdW, electrostatic and total interaction energies (EvdW, Eele and E_inter_) between FRT, FRS, FRI and NA active-site residues a, b.

	**FRT-NA**			**FRS-NA**			**FRI-NA**		

Residue	*E* _vdW_	*E* _ele_	*E* _inter_	*E* _vdW_	*E* _ele_	*E* _inter_	*E* _vdW_	*E* _ele_	*E* _inter_
Arg118	−1.04	−20.01	−21.05	−	−	−	−	−	−
Glu119	−0.40	−30.38	−30.78	−2.75	−65.75	−68.50	−1.82	−56.25	−58.07
Asp151	3.55	−113.02	−109.47	3.39	−124.73	−121.34	−1.22	−70.93	−72.15
Glu227	−0.05	−22.80	−22.85	−1.47	−67.11	−68.58	−0.70	−48.77	−49.47
Ala246	−	−	−	−1.64	−3.41	−5.05	−	−	−
Thr247	−4.46	−1.39	−5.85	−	−	−	−4.35	−0.46	−4.81
Glu276	−0.32	−36.13	−36.45	−0.40	−45.66	−46.06	−0.69	−45.56	−46.25
Glu277	−0.27	−21.88	−22.15	−1.99	−72.05	−74.04	3.25	−108.43	−105.18
Arg292	−	−	−	−	−	−	−1.34	−6.50	−7.84
Asn346	0.20	−16.38	−16.18	−	−	−	−	−	−
Asn347	−4.89	−15.91	−20.80	−3.90	−1.07	−4.97	−	−	−
Arg371	1.05	−48.40	−47.35	0.94	−30.40	−29.46	1.18	−43.88	−42.70
Tyr406	−1.52	−14.99	−16.51	−	−	−	−	−	−
Pro431	−1.78	−2.29	−4.07	−	−	−	−	−	−
Lys432	−	−	−	1.11	−34.44	−33.33	0.83	−29.62	−28.79

a Energy units in kcal mol^−1^;

b *E*_inter_ < −4.00 kcal mol^−1^ are given.

**Table S4.** The vdW, electrostatic and total interaction energies (*E*_vdW_, *E*_ele_ and *E*_inter_) between FRI_dep_, FRI_Ac_, FRI_DMA_ and NA active-site residues*a,b*.

	**FRI _dep_-NA**			**FRI _Ac_-NA**			**FRI _DMA_-NA**		

Residue	*E* _vdW_	*E* _ele_	*E* _inter_	*E* _vdW_	*E* _ele_	*E* _inter_	*E* _vdW_	*E _ele_*	*E* _inter_
Glu119	−1.16	−34.45	−35.61	−4.14	−41.99	−46.13	−1.28	−97.43	−98.71
Ile149	−	−	−	−	−	−	−3.03	−2.78	−5.81
Asp151	−5.76	−19.80	−25.56	−1.52	−74.80	−76.32	−7.84	−155.98	−163.82
Trp178	−	−	−	−2.33	−3.54	−5.87	−	−	−
Glu227	−0.20	−12.64	−12.84	−0.49	−38.62	−39.11	−0.24	−65.95	−66.19
Ala246	−5.69	0.15	−5.54	−	−	−	−	−	−
Glu276	−0.55	−7.29	−7.84	−	−	−	−0.90	−82.62	−83.52
Glu277	−1.37	−19.89	−21.26	−1.83	−32.30	−34.13	−1.33	−94.14	−95.47
Asn346	−	−	−	−	−	−	−0.50	−3.53	−4.03
Asn347	−	−	−	−3.01	−1.15	−4.16	−	−	−
Gly348	−	−	−	−4.61	−7.14	−11.75	−	−	−
Arg371	0.91	−48.85	−47.94	−7.50	−62.66	−70.16	−	−	−
Pro431	−	−	−	−2.97	−2.64	−5.61	−	−	−
Lys432	−	−	−	−0.61	−9.71	−10.32	−	−	−

a Energy units in kcal mol^−1^;

b *E_inter_* < −4.00 kcal mol^−1^ are given.
