# Supplementary figures and images for: Computer-Based De Novo Designs of Tripeptides as Novel Neuraminidase Inhibitors
Source: Int J Mol Sci. 2010 Dec 1;11(12):4932–51. doi: 10.3390/ijms11124932 (PMC3100827; doi:10.3390/ijms11124932)

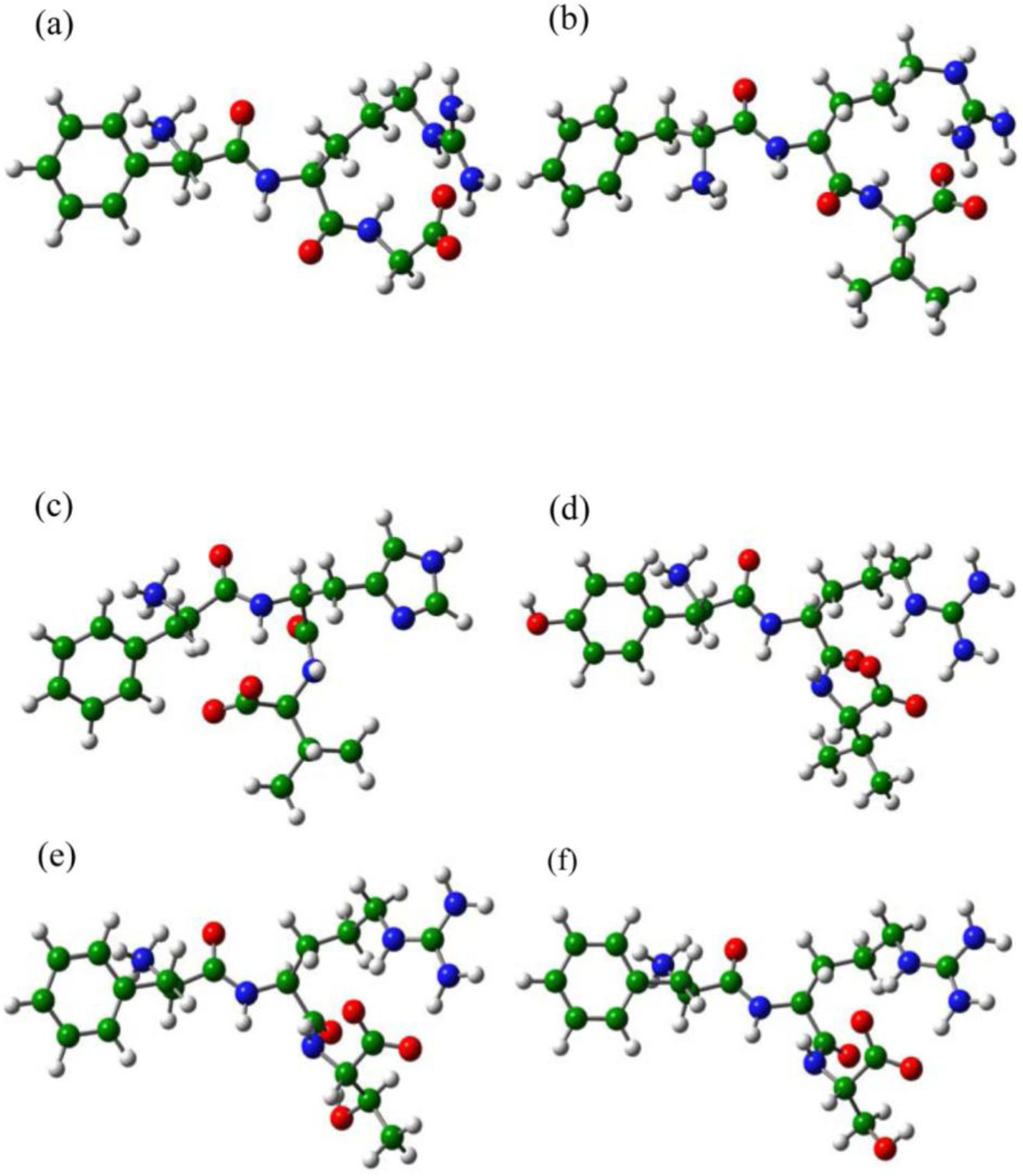

Supplement: Figure S1. — Optimized tripeptide structures at B3LYP/6-31G(d,p) level of theory: (a) FRG; (b) FRV; (c) FHV; (d) YRV; (e) FRT; (f) FRS; (g) FRI; (h) FRIdep; (i) FRIAc and (j) FRIDMA. [file ijms-11-04932f8a.tif]

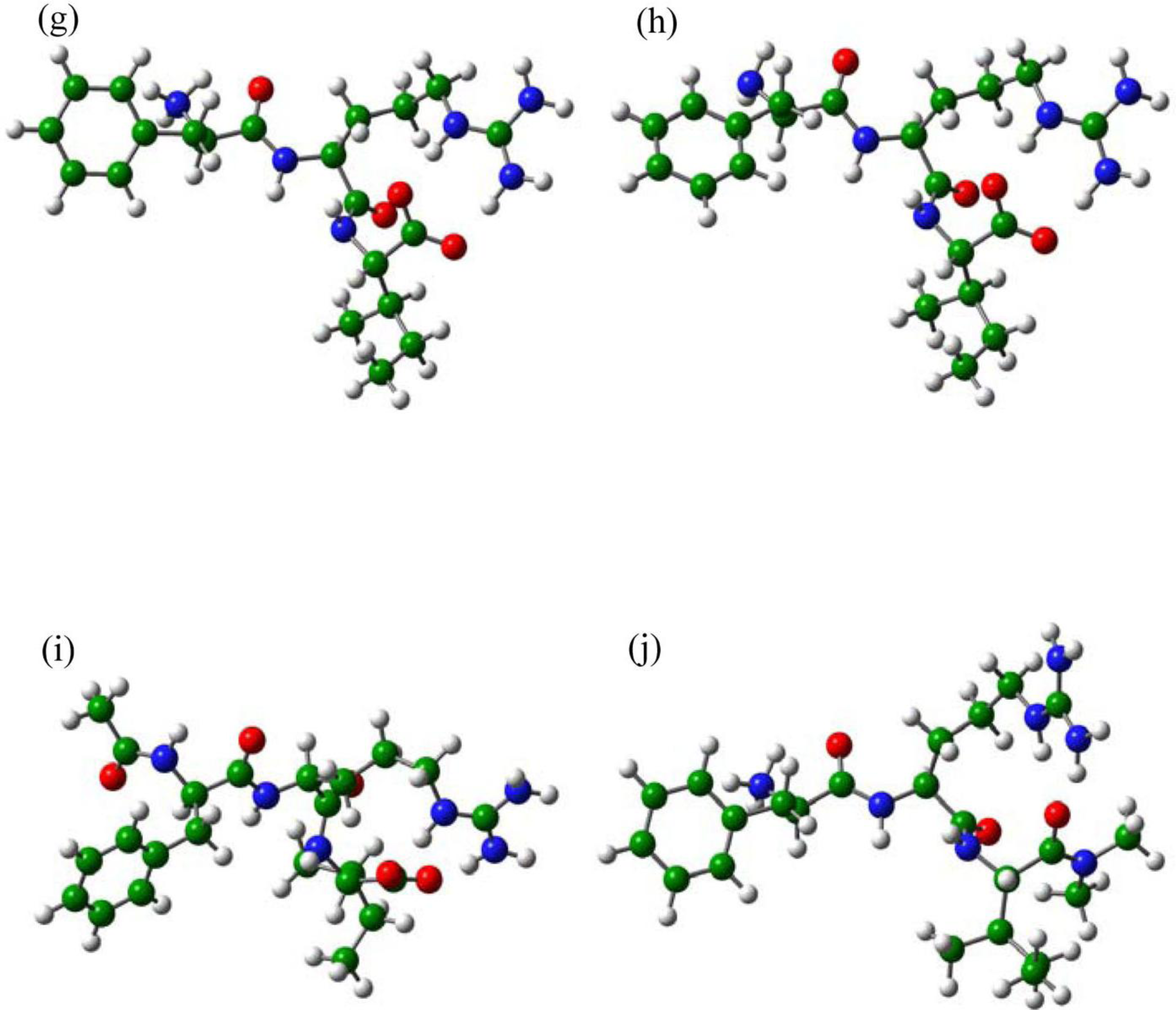

Supplement: Figure S1. — Optimized tripeptide structures at B3LYP/6-31G(d,p) level of theory: (a) FRG; (b) FRV; (c) FHV; (d) YRV; (e) FRT; (f) FRS; (g) FRI; (h) FRIdep; (i) FRIAc and (j) FRIDMA. [file ijms-11-04932f8b.tif]
